# Acaricidal and insecticidal activity of essential oils obtained from the aerial parts of three Mexican *Bursera* species

**DOI:** 10.1007/s11356-023-30895-w

**Published:** 2023-11-17

**Authors:** Felix Krengel, Roman Pavela, Fidel Ocampo-Bautista, Patricia Guevara-Fefer

**Affiliations:** 1 Facultad de Ciencias, Universidad Nacional Autónoma de México (UNAM), Av. Universidad 3000, Circuito Exterior s/n, Alcaldía Coyoacán, Ciudad Universitaria, C.P. 04510 Mexico City, Mexico; 2 Crop Research Institute, Drnovska 507, 161 06, Prague 6, Prague, Czech Republic; 3 Department of Plant Biotechnology, College of Life Sciences and Biotechnology, Korea University, Seoul, 02841 Republic of Korea; 4 Facultad de Ciencias Biológicas, Universidad Autónoma del Estado de Morelos (UAEM), Cuernavaca, Morelos Mexico

**Keywords:** Acaricide, *Bursera glabrifolia*, *Bursera lancifolia*, *Bursera linanoe*, Essential oil, Insecticide, Integrated pest management (IPM), Pesticide

## Abstract

In search of new sustainable biopesticides, we determined the phytochemical profiles, acaricidal and insecticidal properties of EOs distilled from the aerial parts of three Mexican *Bursera* species. Results were obtained by GC–MS analysis and three different bioassays, indicating that the EO of *Bursera glabrifolia* exhibited high relative abundancies of α-pinene, β-myrcene, and α-phellandrene, as well as promising pesticidal activity against *Spodoptera littoralis* larvae (LD_50,90_ = 32.4, 107.2 µg/larva), and *Musca domestica* (LD_50,90_ = 23.2, 103.2, and 13.5, 77.4 µg/female or male adult, respectively) and *Tetranychus urticae* adults (LD_50,90_ = 7.4, 30.3 µg/cm^2^). The *Bursera lancifolia* and *Bursera linanoe* samples contained mainly D-limonene or linalyl acetate and linalool, respectively, and showed generally less potent pesticidal properties (*S*. *littoralis* larva, LD_50,90_ = 45.4, 154.4 and 52.2, 158.7 µg/larva, respectively; female *M*. *domestica* adult, LD_50,90_ = 69.2, 210.9 and 45.1, 243.8 µg/female adult, respectively; *T*. *urticae* adults, LD_50,90_ = 20.7, 90.5 and 17.5, 71.4 µg/cm^2^, respectively). However, the EO of *B*. *linanoe* exhibited an especially pronounced activity against male *M. domestica* adults (LD_50,90_ = 10.6, 77.2 µg/male adult). Our findings prove the pesticidal potential of Mexican *Bursera* species in the context of integrated pest management (IPM) and highlight the importance of conducting further research to elucidate both the active principles and possibly existing synergistic effects.

**Supplementary Information:**

The online version contains supplementary material available at 10.1007/s11356-023-30895-w.

## Introduction

The use of insecticides represents a central element of modern agriculture, in general, and of pest mitigation in particular. Notwithstanding, the widespread application of synthetic formulations in conventional cropping systems all over the world poses serious threats to the environment and human health, which are further exacerbated in tropical climate zones. The development of ideally innocuous bioinsecticides within the framework of integrated pest management (IPM) is therefore of the utmost importance for meeting the United Nations (UN) 17 Sustainable Development Goals (SDG) by 2030 (Struelens and Silvie 2020).

Several studies suggest that these much-needed bioinsecticides might in part be derived from the pan-American *Bursera* Jacq. ex L. (Burseraceae) genus. In this sense, organic leaf extracts from *Bursera copallifera* (Sessé & Moc.) Bullock and *Bursera lancifolia* Engl. were shown to impair the growth and development of *Spodoptera frugiperda* J.E. Smith (Lepidoptera: Noctuidae) larvae (Cárdenas et al. 2012). Moreover, *B*. *copallifera* and *Bursera grandifolia* Engl. leaf and stem bark extracts had a differential impact on *S*. *frugiperda*, dependent on the developmental stage of the latter, as well as the specific plant organ and organic solvent used (Aldana-Llanos et al. 2010). The monoterpenoid-rich essential oil (EO) obtained from the leaves of *Bursera glabrifolia* Engl. or the stems and fruits of *Bursera graveolens* Triana & Planch. exhibited insecticidal activity against *Sitophilus zeamais* Motschulsky (Coleoptera: Curculionidae) in the case of the first species (Villa-Ruano et al. 2018) and ovicidal, insecticidal, or repellent effects against *Acanthoscelides obtectus* Say (Coleoptera: Chrysomelidae), *Calliphora vomitoria* L. (Diptera: Calliphoridae), and *Zabrotes subfasciatus* Boheman (Coleoptera: Chrysomelidae) with regard to the second species (Farina et al. 2021; Jumbo et al. 2022). In addition, the essential oil from the fruits of *B*. *graveolens* also exhibited acaricidal properties against *Rhipicephalus microplus* Canestrini (Ixodida: Ixodidae) larvae (Rey-Valeirón et al. 2017).

Concerning the active principles and mechanisms of action responsible for the pesticidal activity of *Bursera* species, it should be noted that both phenolic compounds and terpenoids occurring within the genus have been highlighted correspondingly, some of which may act as enzyme inhibitors. In accordance with this hypothesis, the aforementioned *B*. *copallifera* and *B*. *lancifolia* samples inhibited *S*. *frugiperda*-derived acetylcholinesterase in vitro (Cárdenas et al. 2012). Similar results were reported in other in vitro or in silico studies evaluating the enzyme inhibitory properties of the essential oil distilled from *B. graveolens* (Eduarte-Saltos et al. 2022; Jumbo et al. 2022). Aqueous leaf extracts from *Bursera simaruba* Sarg., in contrast, inhibited neither α-amylase nor α-glucosidase in vitro (Rodríguez-García et al. 2019).

On a more specific level, EOs represent a phytochemically well-defined fraction within the secondary metabolome of the *Bursera* genus, as they are mainly composed of a variety of mono- and, to a lesser degree, sesquiterpenoids that often contain distinctive functional groups like alcohols, aldehydes, ketones, or lactones (Isman and Machial 2006; Noge et al. 2010). Despite serving as chemical signals between plants and other organisms, EOs can play an important role in allelopathy and plant defense against herbivores and pathogens (Karabörklü and Ayvaz 2023; Park and Tak 2016; Villa-Ruano et al. 2018). For instance, their strong aroma may deter insects from approaching the plant, making it less likely to be a target for feeding or oviposition. On the other hand, direct or indirect contact with EOs—including inhalation or ingestion of volatile monoterpenoids—frequently causes detrimental effects in arthropods (Park and Tak 2016). Terpenoids are generally hydrophobic and thus tend to interact with the lipids and membranes of insects, being cell membrane disruption just one possible consequence of these interactions (Isman and Machial 2006). Furthermore, several terpenoids can alter physiological and behavioral processes of arthropods by interfering with different endocrine, enzyme, and neurotransmitter systems (Park and Tak 2016).

Against this background, we herein tested the EOs of *B*. *glabrifolia*, *B*. *lancifolia*, and *Bursera linanoe* (La Llave) Rzed., Calderón & Medina with respect to their pesticidal activity. The distribution of these three Mexican *Bursera* species is closely associated with the country’s tropical and subtropical dry forests (García-García et al. 2022; Hernández-Pérez et al. 2011). While *B*. *glabrifolia* shows a broader climatic tolerance, but generally prefers rather temperate climate zones, the occurrence of *B*. *lancifolia* and *B*. *linanoe* is restricted to a narrower, high temperature range (Hernández-Pérez et al. 2011). Like many other species of the genus, *B*. *glabrifolia* and *B*. *linanoe* have been employed as natural sources for wood and the aromatic resin *copal* (Blancas et al. 2022; García-García et al. 2022; Gigliarelli et al. 2015; Hernández-Pérez et al. 2011). Production of the latter, in particular, for ritualistic and medicinal use, usually in the form of an incense, dates back to the Pre-Columbian era (Abad-Fitz et al. 2020; Blancas et al. 2022). Today, *copal* is primarily obtained by communities located in central and southern Mexico, often by combining the exploitation of wild and agrisilviculturally managed populations. An average adult specimen of *B*. *glabrifolia* produces an estimated 260 to 280 g of resin. Moreover, in some regions, *B*. *linanoe* has been subjected to human selection in order to improve *copal* yield (Abad-Fitz et al. 2020). Being the resin’s volatile fraction, the EO of this species has also been utilized for commercial purposes (Gigliarelli et al. 2015; Hernández-Pérez et al. 2011). Paradoxically, the cultivation and subsequent processing of *B*. *linanoe* on an industrial scale has only been seen outside of Mexico. The species’ fruit-distilled EO has been particularly valued in India, where it constitutes an important ingredient of perfume formulations (Becerra and Noge 2010; Gigliarelli et al. 2015).

Two insect (*Musca domestica* L. [Diptera: Muscidae] and *Spodoptera littoralis* Boisduval [Lepidoptera: Noctuidae]) and one arachnid (*Tetranychus urticae* C.L. Koch [Trombidiformes: Tetranychidae]) model organisms were selected for the biopesticide assays. The first species is a notorious disease vector, as houseflies can transmit pathogens to humans and animals. This makes them a significant health hazard, potentially spreading viral, bacterial, fungal, and parasitic diseases (Khamesipour et al. 2018). *S. littoralis* is a polyphagous pest, and the larvae of this moth can cause extensive damage by chewing on leaves, stems, and fruits (Hilliou et al. 2021). *T*. *urticae* is a rapidly reproducing mite that feeds on the sap of plants, causing stippling on leaves and reduced plant growth (Santamaria et al. 2020). Severe infestations of *S*. *littoralis* or *T*. *urticae* frequently lead to reduced crop yield and quality. Both species have developed resistance to several chemical pesticides, making their control through traditional chemical means challenging (Hilliou et al. 2021; Santamaria et al. 2020).

Considering that none of these species-specific plant vs. arthropod combinations has been investigated before in an IPM-related context, our findings contribute to determining the pesticidal potential of not only *B. glabrifolia*, *B. lancifolia*, and *Bursera linanoe* but also the *Bursera* genus as a whole.

## Materials and methods

### Plant material

Twigs, leaves, and fruits from *Bursera glabrifolia* Engl. (two individuals, both about 5 m in height), *Bursera lancifolia* Engl. (two individuals, both about 7 m in height), and *Bursera linanoe* (La Llave) Rzed., Calderón & Medina (three individuals, between 4 and 7 m in height) were collected in La Tigra, Puente de Ixtla, Morelos, México (18° 29′ 50.7′′ N 99° 20′ 02.4′′ W, 18° 30′ 18.0′′ N 99° 20′ 21.5′′ W, and 18° 32′ 22.6′′ N 99°19′ 59.8′′ W, respectively) in September of 2021 (Fig. S1). The species were taxonomically determined by Fidel Ocampo Bautista (Universidad Autónoma del Estado de Morelos, UAEM), and voucher specimens were deposited with the Herbarium (HUMO) of the Centro de Investigación en Biodiversidad y Conservación (CIBYC), UAEM (voucher numbers 31061 to 31063). All plant names were checked with http://www.worldfloraonline.org.

### Extraction and phytochemical analysis of essential oils

EOs were obtained by steam distillation on a semi-industrial scale, followed by separating the resulting mixture’s hydrophobic and hydrophilic phases in a separatory funnel. The starting material consisted of 10, 12, and 13 kg of pooled aerial parts from *B*. *glabrifolia*, *B*. *lancifolia*, and *B*. *linanoe*, respectively, leading to the retrieval of 10, 28, and 30 ml of concentrated EOs, respectively (Fig. S1).

The EOs were dissolved in dichloromethane (1 mg/mL) and analyzed using an Agilent 7890B/5977 A GC/MSD with a HP-5 ms (30 m × 250 µm × 0.25 µm) capillary column (EI-mass spectra recording at 70 eV after 3 min of solvent delay; injector in split mode (10:1) at 280 °C with automatic injection of 1 µl aliquots; ramp temperature program from 40 to 310 °C at 8 °C/min; helium carrier gas at 1 ml/min). Peaks were identified by comparison with reference mass spectra of the NIST Mass Spectral Search Program for the NIST/EPA/NIH Mass Spectral Library (Version 14, build May, 2014). The results of this first identification step were then verified by calculating the Kovats retention index of each peak.

### Pesticide bioassays

#### Arthropods

Arthropods were obtained from established laboratory colonies, reared under controlled conditions for more than 20 generations. Uniform larvae of *Spodoptera littoralis* Boisduval (Lepidoptera: Noctuidae) (3rd instar, mean larval weight 12 ± 3 mg), as well as adults of *Musca domestica* L. (Diptera: Muscidae) (males and females, 3 to 5 days old) and *Tetranychus urticae* C.L. Koch (Trombidiformes: Tetranychidae) (females, 1 to 3 days old) were selected for the experiments (Fig. S2). The rearing techniques used for each species have recently been described by Benelli et al. (2019). All species were maintained at 25 ± 1 °C, 70 ± 3% RH and a 16:8 h (L:D) photoperiod. All experiments described below were carried out under the same conditions.

#### Pesticidal activity on target arthropods

In order to evaluate the neurotoxic properties of the EOs, the latter was topically applied on the pronota of *M*. *domestica* adults (both sexes) and *S*. *littoralis* larvae as follows: The EOs were dissolved in acetone (Sigma-Aldrich, Germany) and differentially diluted to obtain a series of test doses (5, 10, 20, 40, 80, 100, 120, 150, and 200 µg/*M*. *domestica* adult and 20, 40, 80, 120, 160, 200, 225, and 250 µg/*S*. *littoralis* larva), of which 1 µL was applied on each carbon dioxide-anesthetized insect using a micro-electric applicator. The control groups were treated with 1 µL of acetone only. Each single replication comprised 20 adults or larvae. After treatment application, the insects were moved to rearing containers (15 × 12 × 8 cm) presenting a perforated lid and containing the respective routine food.

The assays focusing on *T*. *urticae* adults were carried out on blackberry (*Rubus fruticosus* L.) leaf discs (1 cm^2^). The EOs were dissolved in acetone and differentially diluted to obtain a series of test doses consisting of 5, 10, 20, 40, 60, 80, and 100 µg/cm^2^ per sample. A total of 10 µl volumes of the respective solutions were then uniformly applied to the leaf discs using an automatic pipette. The control group was treated with the same volume of pure acetone. Following treatment application, the discs were placed in Petri dishes (5 cm) containing a solid agar layer (0.3-cm thick) on the bottom. The solvent was allowed to evaporate prior to the transfer of 10 T*. urticae* females (1 to 2 days old) onto each of the leaf discs’ treated sides using a fine brush. The Petri dishes were placed in a growth chamber and checked 24 h after treatment application in order to determine the number of dead adults, whereat death was indicated by a lack of response to prodding with forceps. Mortality was assessed 24 h after treatment application. Arthropods that did not exhibit any movement in response to mechanical stimuli were considered dead. These experiments were replicated four times.

#### Statistical analysis

The observed mortality was corrected by Abbott’s formula (Abbott 1925). The dose-mortality data was then subjected to probit analysis in order to estimate the median and 90% lethal doses (LD50 and LD90, respectively) for each treatment, including the associated 95% confidence intervals (CI95; Finney 1971).

## Results and discussion

### Phytochemical composition of essential oils

As expected, most compounds determined by GC–MS turned out to be monoterpenoids. The EO of *B*. *glabrifolia* exhibited the greatest diversity of this substance group, i.e., 14 monoterpenoids in addition to seven sesquiterpenoids. In terms of relative abundance, α-pinene (37.22% of the total area), β-myrcene (25.46%), and α-phellandrene (10.93%) were found to be the majority compounds, while germacrene D (sesquiterpene, 6.44%), D-limonene (4.72%), β-phellandrene (3.56%), β-pinene (1.82%), *o*-cymene (1.66%), caryophyllene (sesquiterpene, 1.20%), and γ-terpinene (1.17%) occurred in smaller yet still significant amounts. With eight monoterpenoids and two sesquiterpenoids, the EO of *B*. *lancifolia* showed a less diverse phytochemical profile that was quantitatively dominated by D-limonene (92.81% of the total area). Cryptone (1.75%), β-myrcene (1.26%), and germacrene D (sesquiterpene, 1.01%) were the most abundant minority compounds. The EO of *B*. *linanoe* contained 11 monoterpenoids and three sesquiterpenoids. Linalyl acetate (68.88% of the total area) and linalool (17.54%) were present in significantly greater quantities than α-terpineol (5.63%), lavandulyl acetate (2.52%), and cis-geranyl acetate (1.04%; Fig. S3; Table 1).

**Table 1:**
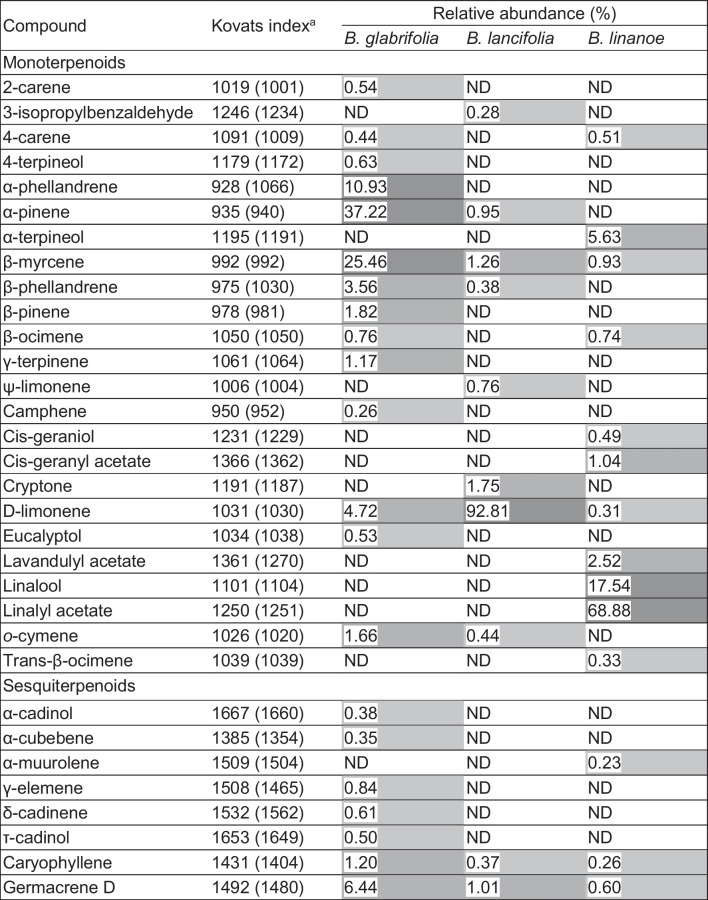
Compounds detected in *Bursera* essential oils according to GC–MS analysis

^a^Experimentally determined and reported (NIST/EPA/NIH Mass Spectral Library, in brackets) Kovats retention index

*ND* not detected

Shades of gray indicate low (< 1%; light gray), medium (1 to 10%; medium gray), and high (> 10%; dark gray) relative abundance

The results were generally in line with the overall chemical composition of the *Bursera* genus (Marcotullio et al. 2018), as the most common volatile monoterpenoids α- (*B*. *glabrifolia*, *B*. *lancifolia*) and β-pinene (*B*. *glabrifolia*), β-phellandrene (*B*. *glabrifolia*, *B*. *lancifolia*), and D-limonene (*B*. *glabrifolia*, *B*. *lancifolia*, *B*. *linanoe*) were found in at least one of the samples analyzed in this study. The highly genus-specific sesquiterpenoids β-caryophyllene and germacrene D were detected in all three samples. In contrast to these rather ubiquitous compounds, other primarily volatile components of the resins or EOs of *Bursera* species serve as distinctive chemical markers of the latter (Gigliarelli et al. 2015). In this context, *B. glabrifolia*-derived EOs have previously been reported (Villa-Ruano et al. 2018; Zúñiga et al. 2005) to contain several of the mono- (4-terpineol, α- and β-pinene, β-myrcene, D-limonene) and sesquiterpenoids (α-cadinol, caryophyllene oxide) listed in Table 1. Although our analyses could not confirm the presence of other registered compounds (monoterpenoids: 1,8-cineole, α-terpinene, α-terpineol, α-thujene, p-cymen-8-ol, p-cymene, linanool, ocimene, sabinene, verbenone; sesquiterpenoids: elemol, nerolidol, spathulenol), they did result in the detection of 13 substances (monoterpenoids: 2- and 4-carene, α- and β-phellandrene, β-ocimene, γ-terpinene, camphene, eucalyptol, *o*-cymene; sesquiterpenoids: α-cubebene, γ-elemene, δ-cadinene, τ-cadinol, germacrene D) that had not previously been reported for the species’ EO. The quantitatively important occurrence of D-limonene in the EO of *B*. *lancifolia* was a key finding of both the present and a previous publication (Zúñiga et al. 2005). In contrast, seven (3-isopropylbenzaldehyde, α-pinene, β-myrcene, β-phellandrene, ψ-limonene, cryptone, *o*-cymene) and two monoterpenoids (4- and α-terpineol), as well as two (caryophyllene, germacrene D) and three sesquiterpenoids (β-eudesmol, agarospirol, and elemol), were only detected in the former or the latter study, respectively. Finally, we confirmed the published findings that *B*. *linanoe* leaves contain significant amounts of linalyl acetate, in addition to the less abundant monoterpenoid linalool and the yet scarcer sesquiterpenoids β-caryophyllene and germacrene D (Noge et al. 2010). Ten other substances (monoterpenoids: 4-carene, α-terpineol, β-myrcene, β-ocimene, cis-geraniol, cis-geranyl acetate, D-limonene, lavandulyl acetate, trans-β-ocimene; sesquiterpenoids: α-muurolene) were exclusively found in our study.

These phytochemical discrepancies may very well be attributed to the different plant organs employed in each research work. For instance, while we obtained the EOs from aerial parts, the three cited publications used either leaves (Noge et al. 2010; Villa-Ruano et al. 2018) or bark (Zúñiga et al. 2005) as source material. Intraspecific, environmentally driven, and/or seasonal variation could be other explanatory factors with regard to the observed metabolic deviations across studies (Villa-Ruano et al. 2018). In this sense, it is important to point out that the EOs analyzed by us were obtained from only two or three specimens per species. Any differences between these samples and the scientific literature could therefore simply be due to intraspecific diversity and should be regarded as such. Concerning the *B*. *linanoe* sample, a rather technical aspect seems to explain quite plausibly why linalool was determined to be the second most abundant compound in our analysis but only a trace component in another article (Noge et al. 2010); linalyl acetate can decompose into linalool by thermal hydrolysis, a process likely taking place during steam distillation, but not cold extraction. Hence, the quantitatively different linalool contents may be a consequence of our reliance on the former, and Noge et al. (2010) opting for the latter, technique to extract volatile compounds from the aerial parts of *B*. *linanoe*.

### Pesticidal activity of essential oils

The EO of *B*. *glabrifolia* exhibited the strongest pesticidal activity against *S*. *littoralis* larvae (LD_50,90_ = 32.4, 107.2 µg/larva; Table 2), as well as *M*. *domestica* (LD_50,90_ = 23.2, 103.2, and 13.5, 77.4 µg/female or male adult, respectively; Table 3) and *T*. *urticae* adults (LD_50,90_ = 7.4, 30.3 µg/cm^2^; Table 4). The *B*. *lancifolia* and *B*. *linanoe* samples proved to have less potent, albeit still effective, pesticidal properties. The respective effects were of a generally similar magnitude (LD_50,90_ = 45.4, 154.4 and 52.2, 158.7 µg/larva, respectively; Table 2; LD_50,90_ = 69.2, 210.9 and 45.1, 243.8 µg/female adult, respectively; Table 3; LD_50,90_ = 20.7, 90.5 and 17.5, 71.4 µg/cm^2^, respectively; Table 4), except for the EO of *B*. *linanoe*, which showed a particularly pronounced activity against male *M*. *domestica* adults (LD_50,90_ = 10.6, 77.2 µg/male adult; Table 3).

**Table 2:** Insecticidal activity of *Bursera* essential oils against *Spodoptera littoralis* 3rd instar larvae

Species	LD_50_ (µg/larva)	CI95	LD_90_ (µg/larva)	CI95	*X*^2^	df	*p*-value
*B*. *glabrifolia*	32.4	22.1–38.8	107.2	90.5–126.1	0.912	4	0.923
*B*. *lancifolia*	45.4	39.9–57.1	154.4	132.8–248.8	2.712	4	0.607
*B*. *linanoe*	52.2	45.5–59.2	158.7	142.8–296.8	0.855	4	0.931

*LD*_*50,90*_ lethal dose killing 50% and 90%, respectively, of the exposed insect population; *CI95* 95% confidence interval; *X*^2^ chi-square; *df* degrees of freedom; *p*-values ≥ 0.05, not significant

**Table 3:** Insecticidal activity of *Bursera* essential oils against *Musca domestica* adults

Species	Stage	LD_50_ (µg/adult)	CI95	LD_90_ (µg/adult)	CI95	*X*^2^	df	*p*-value
*B*. *glabrifolia*	Female	23.2	18.9–28.3	103.2	81.3–137.6	3.341	8	0.899
	Male	13.5	10.7–18.3	77.4	55.1–80.8	1.239	7	0.991
*B*. *lancifolia*	Female	69.2	52.8–82.6	210.9	198.7–328.5	2.854	4	0.122
	Male	45.8	30.7–58.6	231.8	211.5–298.7	1.785	7	0.398
*B*. *linanoe*	Female	45.1	31.8–56.9	243.8	221.9–292.7	1.128	7	0.351
	Male	10.6	8.2–13.9	77.2	54.2–79.9	1.583	8	0.991

*LD*_*50,90*_ lethal dose killing 50% and 90%, respectively, of the exposed insect population; *CI95* 95% confidence interval; *X*^2^ chi-square; *df* degrees of freedom; *p*-values ≥ 0.05, not significant

**Table 4:** Acaricidal activity of *Bursera* essential oils against *Tetranychus urticae* adults

Species	LD_50_ (µg/cm^2^)	CI95	LD_90_ (µg/cm^2^)	CI95	*X*^2^	df	*p*-value
*B*. *glabrifolia*	7.4	5.7–9.8	30.3	22.2–41.8	0.592	3	0.898
*B*. *lancifolia*	20.7	16.3–27.1	90.5	62.5–156.7	0.846	5	0.974
*B*. *linanoe*	17.5	13.6–23.4	71.4	47.3–137.2	0.213	4	0.994

*LD*_*50,90*_ lethal dose killing 50% and 90%, respectively, of the exposed arachnid population; *CI95* 95% confidence interval; *X*^2^ chi-square; *df* degrees of freedom; *p*-values ≥ 0.05, not significant

A further evaluation of the CI95 determined by probit analysis indicated that the pesticidal effects of the *B*. *glabrifolia* sample were significantly greater than those of the other two species’ EOs, which did not differ from each other (Tables 2, 3, and 4). The only exception consisted in the *B*. *lancifolia* sample exhibiting a significantly lower potency against male *M*. *domestica* adults in this regard than the non-differing EOs of *B*. *glabrifolia* and *B*. *linanoe* (Table 3).

Only two relevant studies referring to the insecticidal properties of the three *Bursera* species could be found in the scientific literature. The abovementioned leaf EO of *B*. *glabrifolia* proved to be effective against *S*. *zeamais* adults (Villa-Ruano et al. 2018), whereas terpenoid-containing leaf extracts of *B. lancifolia* showed effects on the larval growth and development of *S*. *frugiperda* (Cárdenas et al. 2012). In the case of *B*. *linanoe*, comparable publications were not available.

From an applied point of view, all three EOs tested could potentially be used as active ingredients in commercial formulations against *S*. *littoralis*, *M*. *domestica*, and/or *T*. *urticae* at a maximal concentration of 50 g/L (5%), given that most of the determined LD_50_ were lower than 50 µg/insect or cm^2^. An indirect comparison of our results with those obtained in other studies under virtually identical experimental conditions (Pavela et al. 2022) helped to further contextualize the foregoing conclusion. For instance, both the EO of *Myrothamnus moschatus* (Baill.) Baill. and the commercially available pesticide Rock Effect, derived from the oil of *Pongamia pinnata* L. (Pierre), were associated with LD_50,90_ of greater, similar, or at least not overwhelmingly smaller magnitude than the values corresponding to the EO of *B. glabrifolia*. While Rock Effect exhibited a higher potency against *S*. *littoralis* (LD_50,90_ = 18.2, 28.6 µg/larva) than the *M. moschatus* (LD_50,90_ = 35.6, 79.2 µg/larva) and the *B*. *glabrifolia* samples (LD_50,90_ = 32.4, 107.2 µg/larva), only the latter two exhibited acute toxicity on female *M*. *domestica* specimens (LD_50,90_ = 22.7, 109.6 and 23.2, 103.2 µg/larva, respectively). *T*. *urticae* was most affected by the *M*. *moschatus* EO (LD_50,90_ = 1.2, 3.3 µg/cm^2^), followed by Rock Effect (LD_50,90_ = 5.8, 10.1 µg/cm^2^) and the *B*. *glabrifolia* sample (LD_50,90_ = 7.4, 30.3 µg/cm^2^). Although the EOs of *B*. *lancifolia* and *B*. *linanoe* appeared to have significantly less potent pesticidal properties against the three model organisms than the *M. moschatus* and Rock Effect samples, they could still hold value in practical terms, particularly when taking into account sublethal doses with a possible impact on population density (Pavela and Benelli 2016).

### Interpreting the essential oil’s biological activity on the basis of their phytochemical profiles

Of the three EOs evaluated, the one obtained from *B*. *glabrifolia* exhibited not only the most pronounced pesticidal activity but also the greatest diversity of both mono- and sesquiterpenoids. Moreover, it represented the only sample displaying substantial relative abundances of α-pinene, β-myrcene, and α-phellandrene. It is therefore plausible to assume that the latter three substances, possibly in synergy with several minority compounds, contribute importantly to the EO’s pesticidal properties. In contrast, the generally lower biological activity of the *B*. *lancifolia* sample, in combination with D-limonene being its sole majority compound, suggests that this monoterpene causes comparatively weaker pesticidal effects, at least considering the experimental models employed in this study. The EO of *B*. *linanoe* proved to be particularly effective against male—but not female—*M*. *domestica* adults, possibly indicating rather selective insecticidal properties of the sample’s main constituents, linalyl acetate, and linalool. It should be mentioned that the acaricidal and insecticidal activity of α-pinene, β-myrcene, α-phellandrene, D-limonene, and linalool, as well as α-terpineol, germacrene D, and several other of the minority compounds listed in Table 1 has been documented in previous research works (Karabörklü and Ayvaz 2023; Noge and Becerra 2009), although none of the latter involved any of the three model organisms used in our study. The reader may refer to Karabörklü and Ayvaz (2023) for a broad review of the topic.

From an evolutionary perspective, the diversification of the secondary metabolism of the *Bursera* genus has been partially driven by coevolution with its main herbivores, many of which pertain to the *Blepharida* Chevrolat (Coleoptera: Chrysomelidae) genus. In this regard, the resin produced by virtually all *Bursera* species represents a core element of at least two plant defense mechanisms. First, it has already been noted that several of the mono- and sesquiterpenoids contained in the resin have repellent or toxic properties against arthropods. Second, herbivore damage to leaves, especially to their fine resin canals, can cause the exudate to squirt out and encapsulate insects (Becerra et al. 2009). Most *Bursera* species that present the latter defense mechanism produce comparatively simple mixture of terpenoids with one or few majority compounds, whereas the resin of non-squirting species is often more complex in this respect (Becerra et al. 2001, 2009). Interestingly, this general rule seems to be in accordance with our results, as the EO of the squirting species *B*. *lancifolia* (Becerra et al. 2009) showed a marked predominance of D-limonene. The greater phytochemical diversity of the *B*. *glabrifolia* sample was in line with the species releasing only small to intermediate amounts of resin upon herbivore attack (Becerra et al. 2001). While no information on the squirt response of *B*. *linanoe* was available, its relatively simple essential oil composition may imply its classification among squirting species. The latter may have evolved because the metabolic cost of resin production increments with the complexity of its terpenoid profile (Becerra et al. 2009). On the other hand, the evolutionary urge of non- (or less) squirting species to biosynthesize more complex terpenoid mixtures as a defense mechanism against insects and other arthropods could explain why, of the three samples analyzed, the EO of *B*. *glabrifolia* proved to be the most effective pesticide.

## Conclusion

We herein investigated the phytochemical profiles and pesticidal properties of EOs distilled from the aerial parts of three Mexican *Bursera* species. All samples contained between eight and 14 monoterpenoids, as well as two to seven sesquiterpenoids, could be associated with a specific chemical composition. The EO of *B*. *glabrifolia* showed high relative abundancies of α-pinene, β-myrcene, and α-phellandrene, while D-limonene proved to be the majority compound in the *B*. *lancifolia* sample. The EO of *B*. *linanoe* contained predominantly linalyl acetate and linalool. Moreover, between nine and 13 terpenoids were detected for the first time in each EO. All samples exhibited effective pesticidal effects against *M*. *domestica*, *S*. *littoralis*, and *T*. *urticae*, with the EO of *B*. *glabrifolia* exhibiting the greatest acaricidal and insecticidal potency.

It is critical to note that these promising results were derived from a small sample size of plants per species and should therefore not be readily generalized. Furthermore, producing sufficient volumes of the evaluated EOs could pose a challenge for their widespread use as biopesticides. Although agrisilvicultural systems oriented at extracting resin from *Bursera* species do exist on a local scale in Mexico and—in the case of *B. linanoe*—on an industrial scale in India, the effort required to adapt and expand these systems according to specific commercial objectives may be considerably greater than that needed to obtain the active principles either from other plants or by synthesis. Future research should therefore focus on experimentally testing our hypothesis that the pesticidal activity observed in this study correlates with the relative abundancies of the respective EOs’ majority compounds. The elucidation of potential synergistic effects should also receive special attention before the evaluation of particular coating techniques, aimed at improving the efficacy of the active principles, is considered (Pavela and Benelli 2016).

### Supplementary Information

Below is the link to the electronic supplementary material.

Supplementary file1 (DOCX 1729 KB)

## Data Availability

The datasets generated during and/or analyzed during the current study are available from the corresponding author on reasonable request.
