# Supplementary material for: Acaricidal and insecticidal activity of essential oils obtained from the aerial parts of three Mexican Bursera species
Source: Environ Sci Pollut Res Int. 2023 Nov 17;30(58):122717–25. doi: 10.1007/s11356-023-30895-w (PMC10724095; doi:10.1007/s11356-023-30895-w)
Supplement: Supplementary file 1 — Supplementary file1 (DOCX 1729 KB) [file 11356_2023_30895_MOESM1_ESM.docx]

**Acaricidal and insecticidal activity of essential oils obtained from the aerial parts of three Mexican *Bursera* species**

Felix Krengel^1^, Roman Pavela^2,3^, Fidel Ocampo-Bautista^4^, Patricia Guevara-Fefer^1*^

^1^Facultad de Ciencias, Universidad Nacional Autónoma de México (UNAM), Av. Universidad 3000, Circuito Exterior s/n, Alcaldía Coyoacán, C.P 04510, Ciudad Universitaria, Ciudad de México, México

^2^Crop Research Institute, Drnovska 507, 161 06, Prague 6, Czech Republic

^3^Department of Plant Protection, Czech University of Life Sciences Prague, Kamycka 129, 165 00 Praha 6, Suchdol, Czech Republic

^4^Facultad de Ciencias Biológicas, Universidad Autónoma del Estado de Morelos (UAEM), Cuernavaca, Morelos, México

^*^Corresponding author: patriciaguevara@ciencias.unam.mx; +52-55-5622-4905

**Abstract**

In search of new sustainable biopesticides, we determined the phytochemical profiles, acaricidal and insecticidal properties of EOs distilled from the aerial parts of three Mexican *Bursera* species. Results were obtained by GC-MS analysis and three different bioassays, indicating that the EO of *B. glabrifolia* exhibited high relative abundancies of α-pinene, β-myrcene, and α-phellandrene, as well as promising pesticidal activity against *Spodoptera littoralis* larvae (LD_50,90_ = 32.4, 107.2 µg/larva), and *Musca domestica* (LD_50,90_ = 23.2, 103.2 and 13.5, 77.4 µg/female or male adult, respectively) and *Tetranychus urticae* adults (LD_50,90_ = 7.4, 30.3 µg/cm^2^). The *B. lancifolia* and *B. linanoe* samples contained mainly D-limonene or linalyl acetate and linalool, respectively, and showed generally less potent pesticidal properties (*S. littoralis* larva: LD_50.90_ = 45.4, 154.4 and 52.2, 158.7 µg/larva, respectively; female *M. domestica* adult: LD_50,90_ = 69.2, 210.9 and 45.1, 243.8 µg/female adult, respectively; *T. urticae* adults: LD_50,90_ = 20.7, 90.5 and 17.5, 71.4 µg/cm^2^, respectively). However, the EO of *B. linanoe* exhibited an especially pronounced activity against male *M. domestica* adults (LD_50,90_ = 10.6, 77.2 µg/male adult). Our findings prove the pesticidal potential of Mexican *Bursera* species in the context of integrated pest management (IPM) and highlight the importance of conducting further research to elucidate both the active principles and possibly existing synergistic effects.

**Keywords:** Acaricide, *Bursera glabrifolia*, *Bursera lancifolia*, *Bursera linanoe*, essential oil, insecticide, integrated pest management (IPM), pesticide

**Acknowledgments**

The authors would like to thank Mayra León Santiago (Laboratorio Nacional de Ciencias para la Investigación y la Conservación del Patrimonio Cultural [LANCIC], Instituto de Química, UNAM) for conducting the GC-MS analyses. Roman Pavela would like to thank the Ministry of Agriculture of the Czech Republic for financial support of the botanical pesticide and basic substances research. Financial support for this work was provided by the Ministry of Agriculture of the Czech Republic (institutional support MZE-RO0423).

**Supplementary Information**

| *a)* 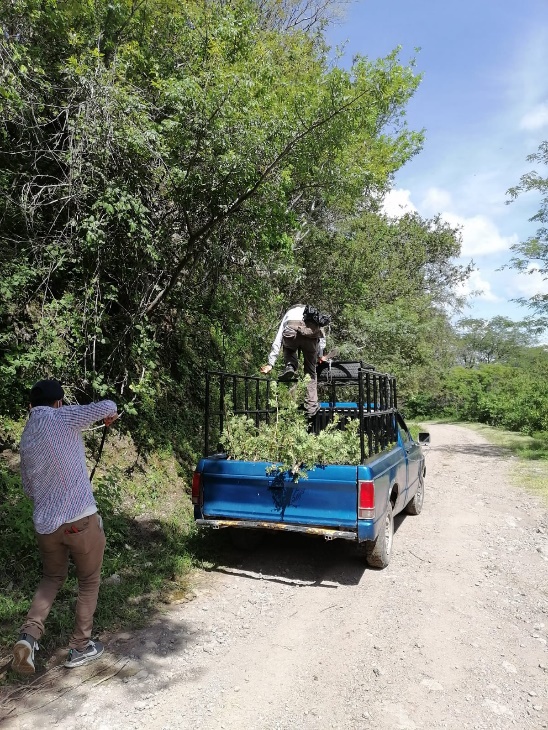 | *b)* 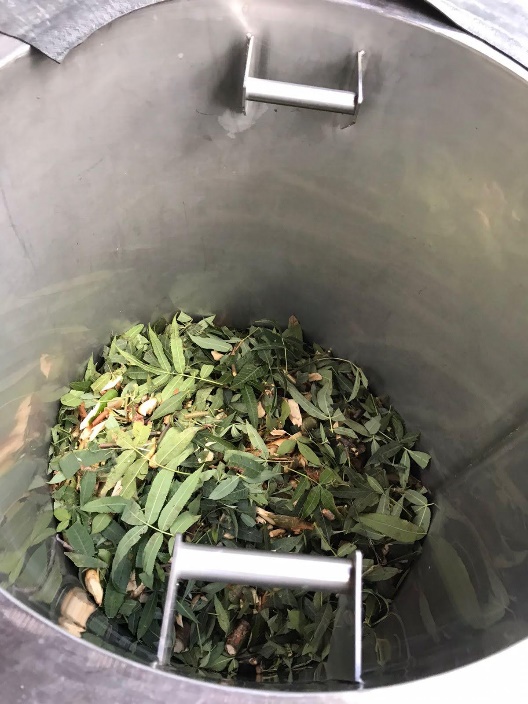 |
| --- | --- |
| *c)* 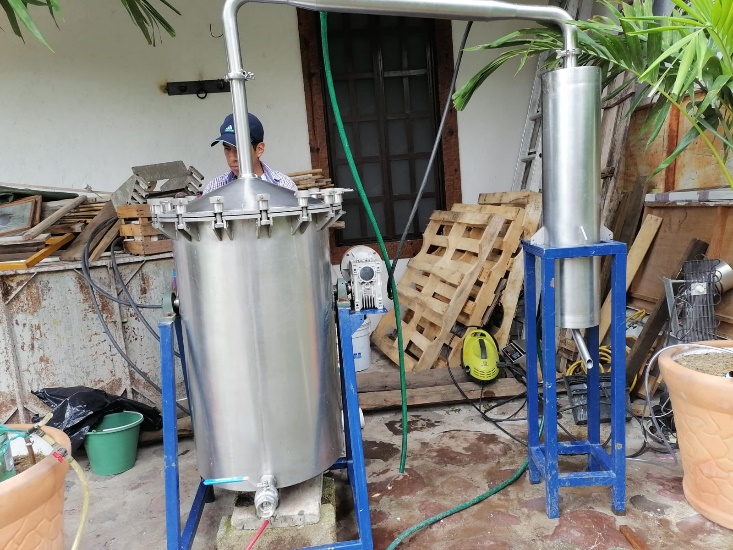 | *d)* 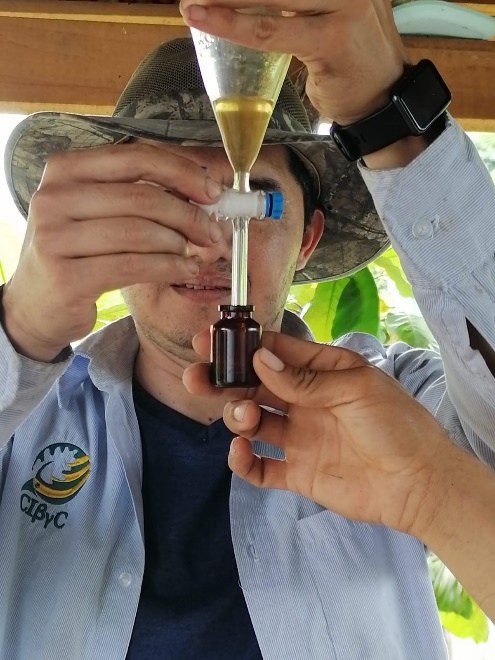 |

**Fig. S1** Process of obtaining essential oils from the aerial parts of *B. glabrifolia*, *B. lancifolia*, and *B. linanoe*: *a)* collecting plant material in the field; *b)* preparing the plant material for steam distillation in a *c)* semi-industrial steam distillation apparatus; *d)* separating the hydrophobic and hydrophilic phases of the distillate in a separatory funnel

| *a)* 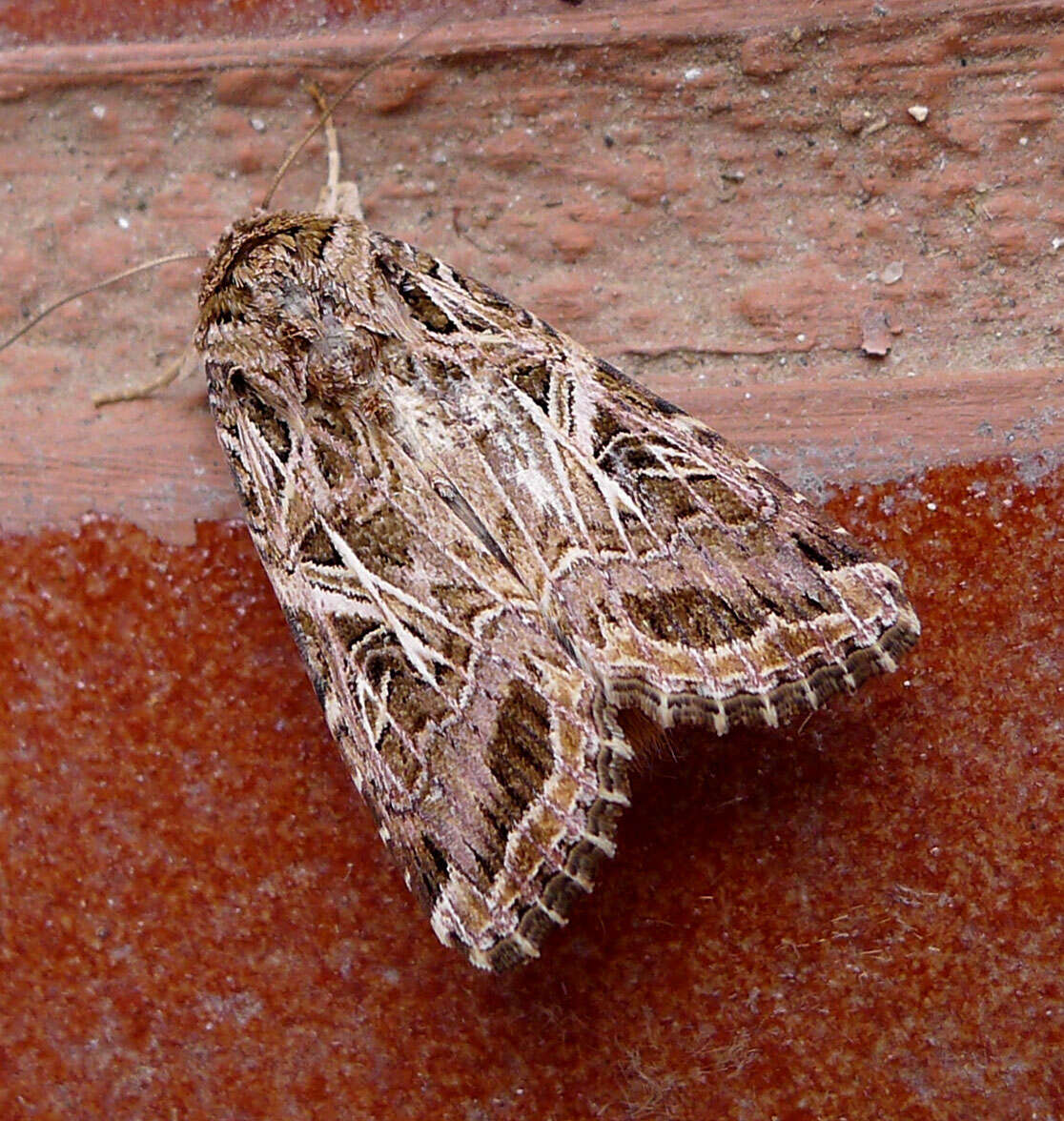 | *b)* 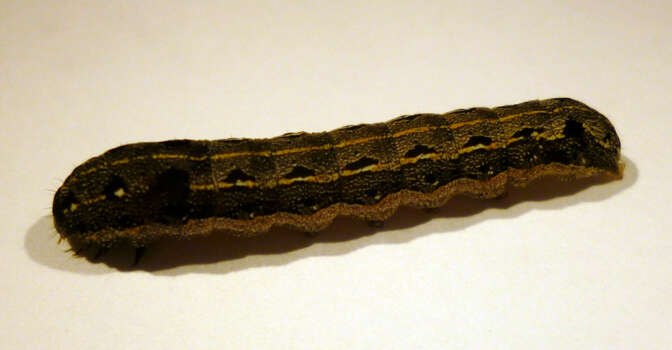 |
| --- | --- |
| *c)* 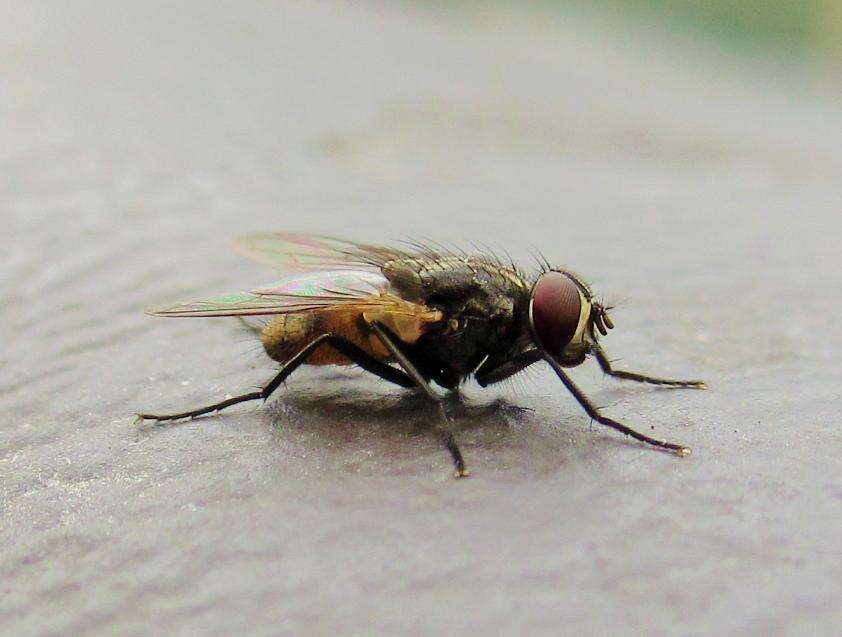 | *d)* 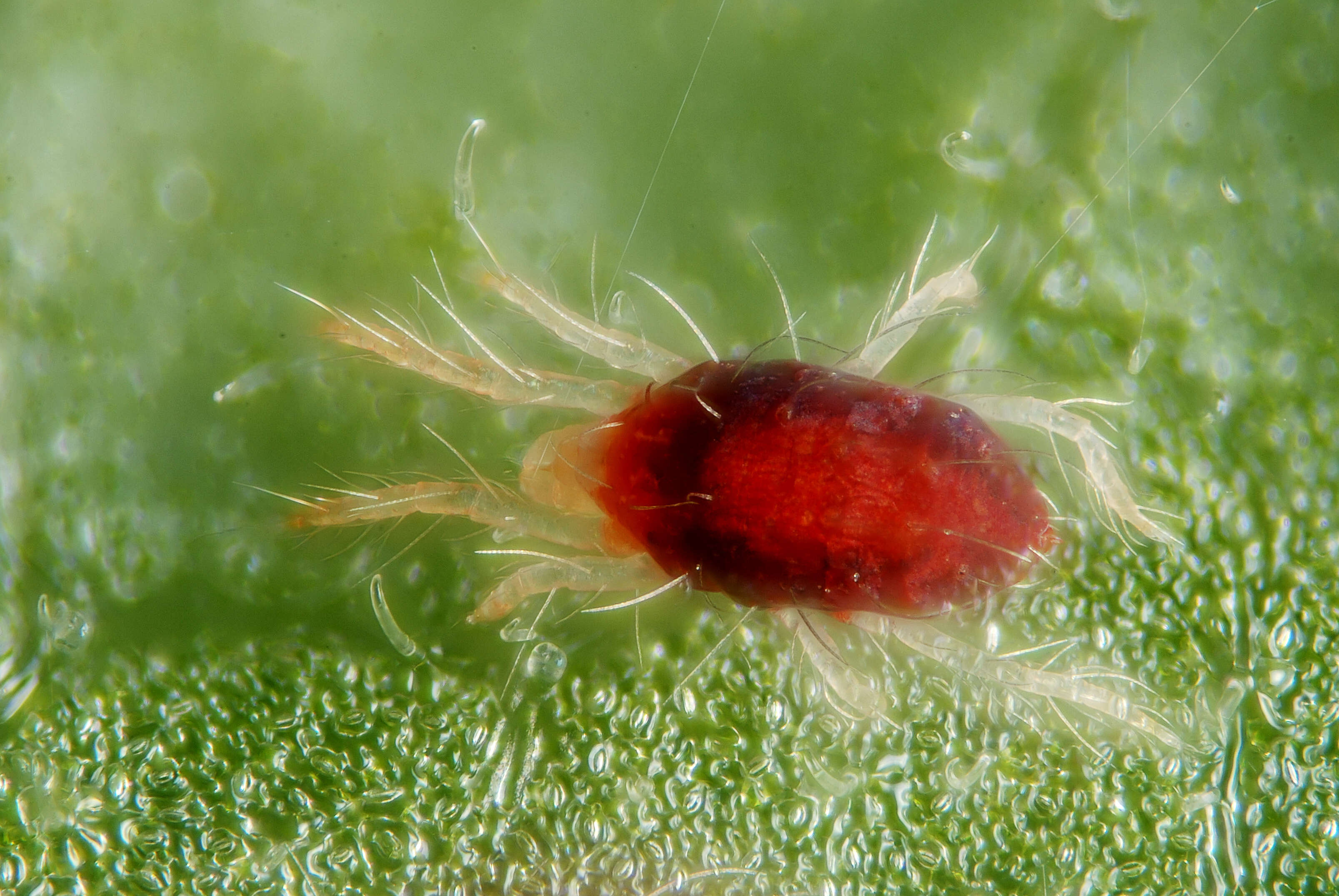 |

**Fig. S2** Model organisms used in the pesticide bioassays: *a)* *Spodoptera littoralis* Boisduval (Lepidoptera: Noctuidae) adult (not used in the bioassays) and *b)* larva (3rd instar larvae, mean larval weight 12 ± 3 mg, were used in the bioassays); *c)* *Musca domestica* L. (Diptera: Muscidae) adult (3 to 5 days old males and females were used in the bioassays); *d)* *Tetranychus urticae* C.L. Koch (Trombidiformes: Tetranychidae) adult (1 to 3 days old females were used in the bioassays)

All images were retrieved from Encyclopedia of Life (https://eol.org/pages/533123/media; https://eol.org/pages/46807345/media; https://eol.org/de/pages/3198999/media).

| *a)* 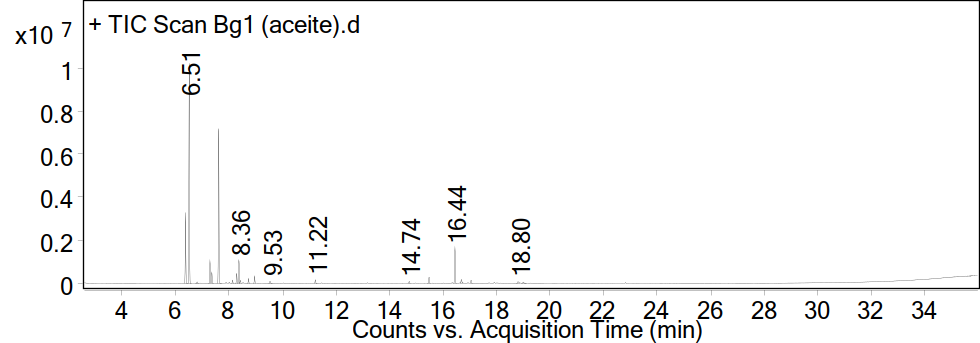 |
| --- |
| *b)* 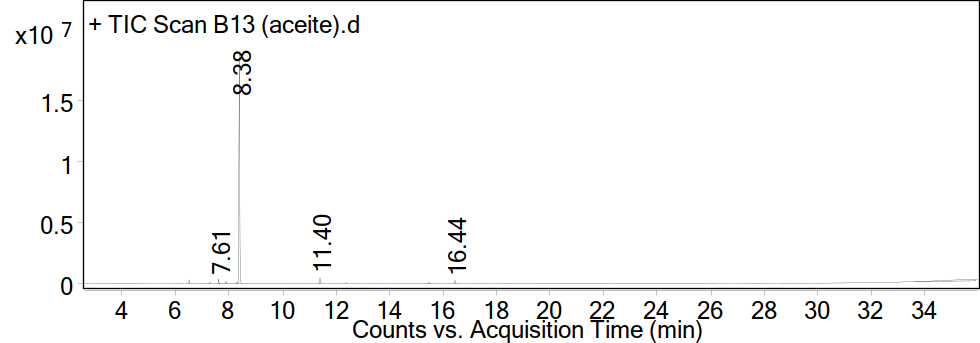 |
| *c)* 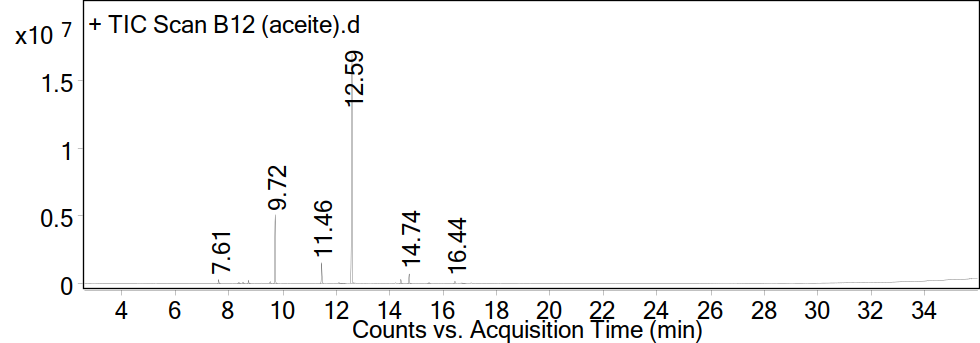 |

**Fig. S3** Experimental chromatograms of the essential oils obtained from the aerial parts of *a) B. glabrifolia*, *b) B. lancifolia*, and *c) B. linanoe*

Retention times (RT) *a)* 6.37 min = α-phellandrene, 6.51 min = α-pinene, 6.80 min = camphene, 7.28 min = β-phellandrene, 7.35 min = β-pinene, 7.61 min = β-myrcene, 8.13 min = 2-carene, 8.28 min = *o*-cymene, 8.36 min = D-limonene, 8.42 min = eucalyptol, 8.73 min = β-ocimene, 8.95 min = γ-terpinene, 9.53 min = 4-carene, 11.22 min = 4-terpineol, 14.74 min = α-cubebene, 15.48 min = caryophyllene, 16.44 min = germacrene D, 16.68 min = γ-elemene, 17.04 min = δ-cadinene, 18.80 min = τ-cadinol, 19.00 min = α-cadinol

RT *b)*: 6.51 min = α-pinene, 7.29 min = β-phellandrene, 7.61 min = β-myrcene, 7.88 min = ψ-limonene, 8.29 min = *o*-cymene, 8.38 min = D-limonene, 11.40 min = cryptone, 12.37 min = 3-isopropylbenzaldehyde, 15.47 min = caryophyllene, 16.44 min = germacrene D

RT *c)*: 7.61 min = β-myrcene, 8.36 min = D-limonene, 8.52 min = trans-β-ocimene, 8.73 min = β-ocimene, 9.53 min = 4-carene, 9.72 min = linalool, 11.46 min = α-terpineol, 12.10 min = cis-geraniol, 12.59 min = linalyl acetate, 14.42 min = cis-geranyl acetate, 14.74 min = lavandulyl acetate, 15.47 min = caryophyllene, 16.44 min = germacrene D, 16.70 min = α-muurolene
